# COVID-19 Mobile Apps in Saudi Arabia: Systematic Identification, Evaluation, and Features Assessment

**DOI:** 10.3389/fpubh.2022.803677

**Published:** 2022-03-18

**Authors:** Nouf Sahal Alharbi, Nada Alsubki, Sara Rasheed Altamimi, Wadi Alonazi, Mochammad Fahlevi

**Affiliations:** ^1^Department of Health Administration, College of Business Administration, King Saud University, Riyadh, Saudi Arabia; ^2^Department of Health Sciences, College of Applied Studies and Community Service, King Saud University, Riyadh, Saudi Arabia; ^3^Public Administration Department, College of Business Administration, King Saud University, Riyadh, Saudi Arabia; ^4^Management Department, BINUS Online Learning, Bina Nusantara University, West Jakarta, Indonesia

**Keywords:** mobile applications, COVID-19, coronavirus, Saudi Arabia, exploratory study

## Abstract

**Background:**

The coronavirus disease 2019 (COVID-19) pandemic is the greatest global health threat in our century at the moment, and the use of mobile health apps has been one digital healthcare strategy adopted for coping with this outbreak.

**Objective:**

This study aims to identify and explore the mobile applications that are currently being utilized for dealing with COVID-19 in Saudi Arabia.

**Methods:**

The applications were selected based on the (Preferred Reporting Items for Systematic Reviews and Meta-Analysis) PRISMA guidelines, then the functionalities were extracted based on the COVID-19 application mind map. Finally, the quality of the apps was assessed using the Mobile Application Rating Scale (MARS) for overall quality, satisfaction, engagement, functionality, aesthetics, and information.

**Results:**

The search identified six applications that were currently being used for COVID-19 which provided the following functionalities: self-assessment, self-isolation, permit for car mobility, prevention guidelines, COVID-19 lab results, call support, identifying nearby facilities, reporting suspected cases, and booking clinic appointments and the COVID-19 test. The findings showed that while most of these features were provided by multiple apps, on the MARS, the overall scores ranged from 3.26 to 3.69 with the apps scoring lower in the areas of satisfaction and engagement and higher in functionalities.

**Conclusion:**

Further steps are needed to unify all these functions in one health app to enhance the users' experience.

## Background

As the coronavirus disease (COVID-19) unfolds, it has become the greatest health crisis in our recent history (1). Since the first case was discovered in December 2019, this virus has spread exponentially to become a global pandemic (2). Without any effective pharmaceutical measures in place, and to limit the spread of the pandemic, many governments have relied on precautionary responses, such as case isolation, social distancing, travel restrictions, and partial or full lockdowns (2, 3). Fortunately, the fight against this disease is in the age of digital technology, and the WHO has issued a list of recommendations for improving population health through the adoption of health information technologies (4).

Mobile applications are among the tools of the digital revolution that allow distance connectivity and which have proven their effectiveness and efficiency in terms of flexibility, functions, and accessibility (5). At present, the rate of smart phone usage is 48% worldwide, and so, this huge user base indicates a growing opportunity for the effective use of mobile applications for preventing, treating, and controlling the pandemic (6, 7). In Saudi Arabia, it was estimated in 2019 that there were more than 40 million mobile cellular subscribers with 95% of them having access to the internet (6). Considering the widespread adoption of smartphones by the Saudi population, the Saudi Ministry of Health (MOH) launched many Health apps for public health purposes (3).

Although the COVID-19 pandemic is a recent phenomenon, previous studies have been conducted to identify and describe several features on more than sixty mobile health applications related to the virus from different countries. The studies found that the market has seen the appearance of numerous mobile applications for tracking and managing the pandemic. Furthermore, the app-market remains disorganized and unregulated in several countries (8–10). However, none of these studies covered any apps from Saudi Arabia nor included an analysis from a national system perspective. To address this gap, our study focuses on Saudi Arabia which is one of the countries committed to digital transformation as one of its national goals aligned with UN's Vision 2030 (11). In this respect, this study, within the selected country, provides an excellent research context for giving a comprehensive and unique assessment of all available mobile applications through the lens of the COVID-19 pandemic.

## Methods

Our study offers three major contributions to the field. First, COVID-19 apps were systematically selected based on the Preferred Reporting Items for Systematic Reviews and Meta-Analysis (PRISMA) guidelines (12). Secondly, the mobile phone applications were evaluated based on the Mobile Application Rating Scale (MARS) (13). Finally, we gathered and extracted information about app functionalities by adopting the COVID-19 app feature mind map (14).

### Finding Relevant COVID-19 Apps

In May 2020, we conducted an extensive review of the mobile health apps in the Saudi Apple iTunes Store and Android Google Play store. The PRISMA guidelines were adopted for this study (12). The app selection process was conducted in three phases. In the first phase, app identification was performed using both the English and Arabic languages using the following search terms - “COVID,” “COVID-19,” “Corona,” “Coronavirus,” “Corona pandemic,” “Corona and self-care,” “COVID-19 and symptoms monitoring,” “self-care and COVID-19,” and “Corona and COVID-19.” Each term was searched in the Android Google Play and Apple Stores.

**Figure 1:**
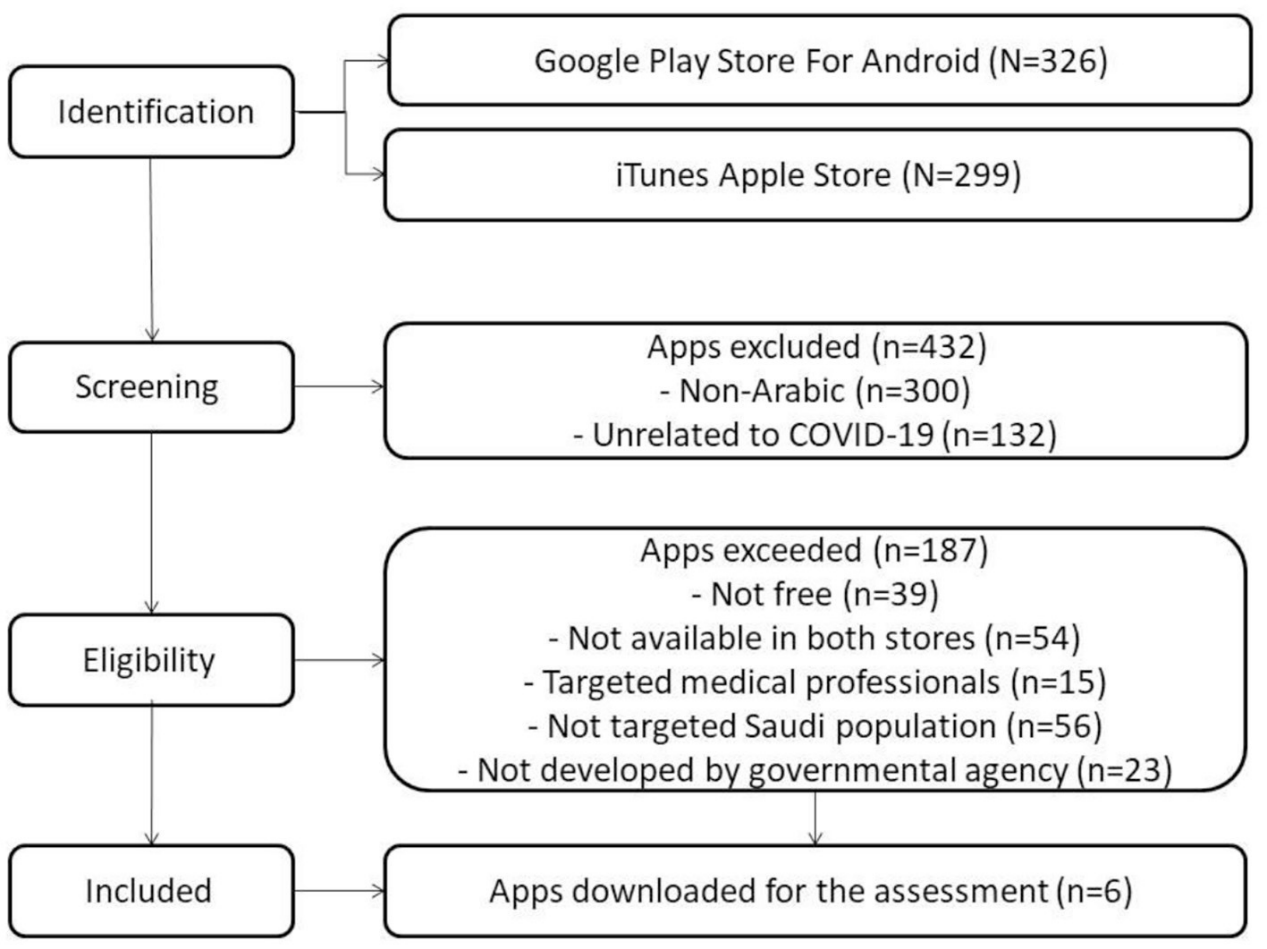
PRISMA flow diagram of app selection.

In the second phase, initial screening was conducted based on the app titles and their full description, and apps were excluded if they were not supported by the Arabic language and not tailored toward service for COVID-19. In the third phase, a set of the criteria was applied to exclude the following apps: those not found in both stores, freely accessible, targeted at medical professional, not aimed at the Saudi population, and not developed by a governmental agency (Figure 1). Finally, a total of six apps was selected, downloaded, reviewed, and assessed as shown in Figure 1.

### Apps Evaluations

We rated and ranked the apps based on the MARS evaluation tool (13) which has three sections: app classification, objective quality, and subjective quality. The instrument consisted of 23 questions designed to help in the analysis of the five scales, namely, the level of entertainment provided, whether it was interesting to use - “Engagement”; the performance and ease of use - “Functionality”; overall visual appeal - “Aesthetics”; quality and quantity of information/accuracy of app description -“Information”; and subjective quality of the mobile applications - “Satisfaction.” The mean score of these subscales was estimated using a 5-point Likert scale (1= inadequate, 2= poor, 3=acceptable, 4= good and 5 = excellent). A group of 22 health informatics specialists was purposively selected as an expert panel to rate the chosen apps. After viewing the education materials for assessing the apps using the MARS tool, each reviewer independently tested and assessed each of the app for at least 10 min. In order to evaluate the MARS scores from the reviewers, the data were entered and analyzed using the IPM statistical package for social sciences (SPSS) version 20.0. The statistical analysis procedure included a separate descriptive analysis of the MARS and the mean for each app.

### Apps Futures and Functionalities Explorations

Two authors, NS and ST, extracted the general information about the apps for descriptive purposes. The data included: the app's name, main aim, developer, size, number of downloads, and star rate. In addition, the researchers identified the apps' general functionalities by adopting the COVID-19 app feature mind map developed by Islam and colleagues (14). This mind map provides an analytical framework developed from a systematic review and analysis of 25 international apps targeted at COVID-19. First of all, the researchers went through the apps' description and documented the stated features independently. The apps were then installed, and the extracted features were verified, and any additional features were explored. After the investigation, the reviewers assigned initial features to the main themes according to Islam‘s mind map. Lastly, the features were then reviewed again by the team and an eventual consensus was ascertained as to what constituted the final themes.

## Results

### COVID-19 Mobile Apps

Our search identified six apps, two of them, Tatmman, and Tawakkalna, were developed specifically for COVID-19, while the other apps provided some features that supported COVID-19 care. The majority were developed by the MOH, while the others were done by the Saudi Red Crescent Authority (SRCA) and the National Information Center (NIC). Login to all of these apps required national/citizenship ID, except for Aseafni. The app's size in megabytes ranged from 25 for Aseafni to 116.2 for the Sehha app. The apps' number of downloads ranged from 100,000+ to 1,000,000+, and the most downloaded apps were Mawid and Tawakkalna, while the star rating ranged from 3.6 for Sehhaty app to 4.7 for both of Mawid and Tawakkalna (Table 1).

**Table 1:** Description of coronavirus disease 2019 (COVID-19)-related apps.

**App name**	**Translated English name**	**Main aim**	**Developer**	**Size (MB)**	**Downloads**	**Star rating**
**Aseafni**	Help me	Emergency services	SRCA	25	500K+	3.7
**Mawid**	Appointment	Booking appointment	MOH	59.6	1M+	4.7
**Sehha**	Health	Medical consultation	MOH	116.2	500K+	4.1
**Sehhaty**	My health	Health services	MOH	52.5	100K+	3.6
**Tatmman**	Rest assured	Self-isolation	MOH	39.5	100K+	3.7
**Tawakkalna**	Let's go	Request curfew passes	NIC	57.5	1M+	4.7

### Evaluation of COVID-19-Related Apps

Table 2 presents the four subscale scores (engagement, functionality, aesthetics, and information), the subjective quality score (satisfaction), and the overall MARS score. Of the four subscales, functionality had the highest scores and engagement the lowest for all the reviewed apps. According to our analysis, the MARS overall scores for all the apps were similar, with the highest rated app being Sehhaty, while the lowest score was for Aseafni (means 3.69, 3.26 out of 5, respectively). However, the estimated scores for satisfaction were below the midpoint xsas the mean scores were <2.7 for all the apps.

**Table 2:** Mobile applications Mobile Application Rating Scale (MARS) rating scores.

**App name**	**Engagement**	**Functionality**	**Aesthetics**	**Information**	**Satisfaction**	**Overall**
**Aseafni**	2.92	3.71	3.37	3.19	2.04	3.26
**Mawid**	3.34	3.81	3.75	3.49	2.55	3.56
**Sehha**	3.46	3.87	3.68	3.49	2.56	3.59
**Sehhaty**	3.63	3.84	3.89	3.56	2.62	3.69
**Tatmman**	3.35	3.76	3.76	3.60	2.40	3.59
**Tawakkalna**	3.35	3.76	3.76	3.60	2.40	3.59

### Functionalities of COVID-19-Related Apps

**Figure 2:**
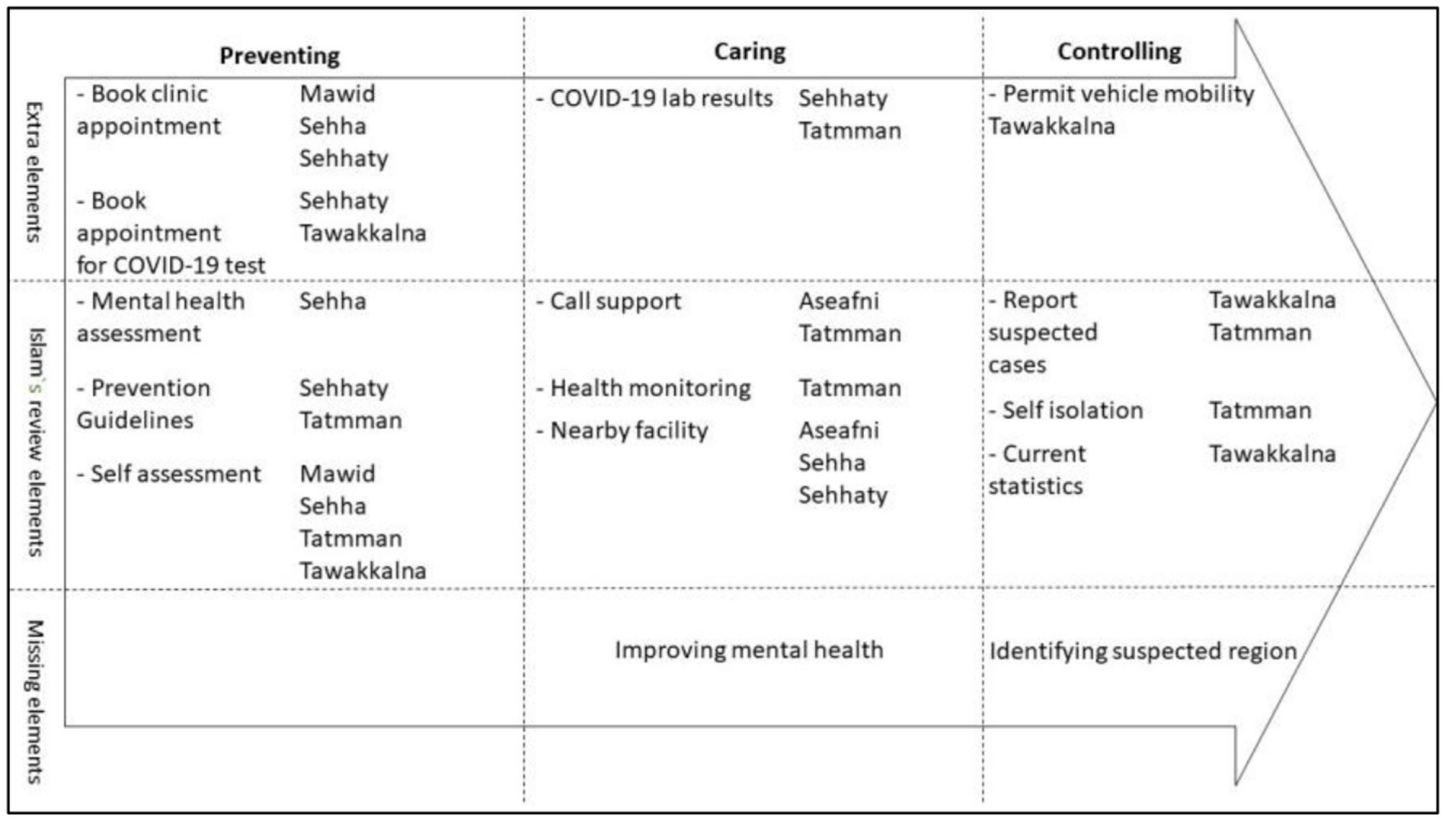
Mapping mobile health app features in accordance with the disease stages and the Islam et al. (14) review.

Plotting the features of the reviewed apps in accordance with the Islam mind map revealed nine main functionalities, namely, mental health assessment, COVID-19 prevention guideline, symptoms checker, communication support, patient monitoring, finding out nearby medical facilities, reporting suspected COVID-19 cases, self-isolation, and the current and statistics about COVID-19 cases. All of these were crossed-matched with the Islam elements. Furthermore, booking an appointment for the clinic and COVID-19 test, obtaining the lab results and getting permission for car mobility were extra functions identified by our study. However, two features, improving mental health for infected cases and identifying suspected regions, were missing from the reviewed apps. Another important result from our analysis was the overlap of some of the apps' functionalities. For example, the COVID-19 self-assessment test provided by Mawid, Sehha, Tatmman, and Tawakkalna and the booking of appointment for a COVID-19 test could be conducted via the Sehhaty and Tawakkalna apps. Figure 2 illustrates the main app features according to the Islam mind map and the COVID-19 stages.

## Discussion

Our study provided a comprehensive and unique analysis of the COVID-19-related apps in Saudi Arabia. To the best of our knowledge, this study is one of the first that explored the main government health app features and assessed their quality in the context of Saudi Arabia. We identified six mobile applications from among the large number of apps developed by the Saudi authorities. We discovered that several functionalities were provided across these apps as well as some features that were not identified in other COVID-19 apps being used internationally. These included the booking of appointment for COVID-19 test, lab results, and persons getting permission for vehicular movement. However, improving mental health and identifying the suspected region with the highest disease rate were missing from all these apps. With regard to the apps' quality, the overall apps' MARS scores in our study were suboptimal. From the results, it seemed that all the apps focused mostly on their functionalities without considering user engagement. However, the variation between these scales was not exclusive to Saudi Arabia but was also observed internationally for other COVID-19 applications (9, 10).

In keeping with previous studies which assessed COVID-19 mHealth apps in developed and developing countries, the raters in our study reported relatively lower technology satisfaction scores (10). In Saudi Arabia, the public had been exposed to a number of apps offering a mix of functions and this might have confused them and, therefore, affected their level of satisfaction. For example, considering that Sehha, Sehhaty, Mawid, and Tetamman were developed by the same organization, digital fragmentation could have resulted from too many apps being created or used (15). In addition, the name of an app should be easy to remember and indicate its functionalities (16). However, in the case of Sehha and Sehhaty, the names are quite similar which could be problematic for the users. Therefore, previous studies have emphasized that the placing of the users' perspectives at the center of the technological advances is crucial in the delivery of healthcare (15, 17).

At a national health care system level, Perle has referred to the app that provides several functionalities as an “all-in-one” app (16). Comparing the current mobile health application status in Saudi Arabia with neighboring countries like the UAE and Oman, previous studies have identified only one COVID-19 app in each region (COVID-19UAE and Tarassud). Furthermore, these apps have scored 4.7 and 4.2, respectively, on the overall MARS (9, 10). In fact, providing the digital services under one platform not only reduces confusion among the users, but it also is cost-effective in the face of a global economic collapse (9). The development of health apps needs to be supported by a governance framework (7, 15, 17, 18) and the Ministry of Interior in Saudi Arabia is a successful example in this regard. This is so because the Ministry provides more than 40 services that fall under several domains (e.g., vehicles, citizens, resident, and other such services) using one platform/application and this has proven to be very successful and effective (19). The Saudi Health Council has the major role in developing a mobile health app policy that can facilitate providing all COVID-19 functions in one app.

Research into mobile applications for COVID-19 is scarce. Our study is the first that assessed apps from a national perspective. Both the MARS and the Islam mind map were found to be very useful in exploring the current status of health apps, and further strengthen the findings of our study (13, 14). However, this study has two limitations that must be taken into consideration for further research. First, the apps surveyed may have been updated to newer versions creating the likelihood that there might be differences in their functionalities. Second, this study did not take into account the end users or the public's perspective of the reviewed apps which might be different when compared to the assessment made by the health informatics experts.

## Conclusion

With regard to the recent COVID-19 pandemic, many mobile applications have been developed to support the public and to improve health outcomes. Within this context, the present paper provides an overview of some of the mobile applications in Saudi Arabia from the perspective of the national health system. We believe that this pandemic has not only provided an opportunity to demonstrate the quality and the functionalities of COVID-19-related apps, but it also allowed for the exploration of the overall digital healthcare system in the country. Therefore, our findings could be a good source of information for our policymakers tasked with the major responsibility for taking the required initiatives to support and guide the creation of mobile health applications.

## Data Availability Statement

The original contributions presented in the study are included in the article/supplementary material, further inquiries can be directed to the corresponding author.

## Ethics Statement

Written informed consent was obtained from the individual(s) for the publication of any potentially identifiable images or data included in this article.

## Author Contributions

NSA and NA conceived of the presented idea. SA and WA developed the theory and performed the computations. MF verified the analytical methods. All authors discussed the results and contributed to the final manuscript.

## Conflict of Interest

The authors declare that the research was conducted in the absence of any commercial or financial relationships that could be construed as a potential conflict of interest.

## Publisher's Note

All claims expressed in this article are solely those of the authors and do not necessarily represent those of their affiliated organizations, or those of the publisher, the editors and the reviewers. Any product that may be evaluated in this article, or claim that may be made by its manufacturer, is not guaranteed or endorsed by the publisher.
